# mTOR-Dependent Oxidative Stress Regulates oxLDL-Induced Trained Innate Immunity in Human Monocytes

**DOI:** 10.3389/fimmu.2018.03155

**Published:** 2019-01-22

**Authors:** Yahya Sohrabi, Sina M. M. Lagache, Lucia Schnack, Rinesh Godfrey, Florian Kahles, Dennis Bruemmer, Johannes Waltenberger, Hannes M. Findeisen

**Affiliations:** ^1^Department of Cardiology I-Coronary and Peripheral Vascular Disease, Heart Failure, University Hospital Münster, Münster, Germany; ^2^Department of Physiology, Cardiovascular Research Institute Maastricht (CARIM), Maastricht, Netherlands; ^3^Department of Internal Medicine I-Cardiology, University Hospital Aachen, Aachen, Germany; ^4^Department of Medicine, Pittsburgh Heart, Lung, Blood, and Vascular Medicine Institute Division of Cardiology, University of Pittsburgh Medical Center (UMPC) and University of Pittsburgh School of Medicine, Pittsburgh, PA, United States; ^5^Medical Faculty, University of Münster, Münster, Germany; ^6^Cells-in-Motion Cluster of Excellence (EXC 1003–CiM), University of Münster, Münster, Germany

**Keywords:** trained innate immunity, monocytes, mTOR, oxidative stress, HIF1α

## Abstract

**Introduction:** Cells of the innate immune system particularly monocytes and macrophages have been recognized as pivotal players both during the initial insult as well as the chronic phase of atherosclerosis. It has recently been shown that oxidized low-density lipoprotein (oxLDL) induces a long-term pro-inflammatory response in monocytes due to epigenetic and metabolic reprogramming, an emerging new concept called trained innate immunity. Changes in the cellular redox state are crucial events in the regulation of many physiologic functions in macrophages including transcription, differentiation and inflammatory response. Here we have analyzed the role of reactive oxygen species (ROS) in regulating this proinflammatory monocyte priming in response to oxLDL-treatment.

**Methods and Results:** Human monocytes were isolated and incubated with oxLDL for 24 h. After 5 days of resting, oxLDL treated cells produced significantly more inflammatory cytokines upon restimulation with the TLR2-agonist Pam3cys. Furthermore, oxLDL incubation induced persistent mTOR activation, ROS formation, HIF1α accumulation and HIF1α target gene expression, while pharmacologic mTOR inhibition or siRNA mediated inhibition of the mTORC1 subunit Raptor prevented ROS formation and proinflammatory priming. mTOR dependent ROS formation was associated with increased expression of NAPDH oxidases and necessary for the emergence of the primed phenotype as antioxidant treatment blocked oxLDL priming. Inhibition of cytosolic ROS formation could also block mTOR activation and HIF1α accumulation suggesting a positive feedback loop between mTOR and cytosolic ROS. Although mitochondrial ROS scavenging did not block HIF1α-accumulation at an early time point (24 h), it was persistently reduced on day 6. Therefore, mitochondrial ROS formation appears to occur initially downstream of the mTOR-cytoROS-HIF1α feedback loop but seems to be a crucial factor that controls the long-term activation of the mTOR-HIF1α-axis.

**Conclusion:** In summary, our data demonstrate that mTOR dependent ROS production controls the oxLDL-induced trained innate immunity phenotype in human monocyte derived macrophages. Pharmacologic modulation of these pathways might provide a potential approach to modulate inflammation, associated with aberrant monocyte activation, during atherosclerosis development.

## Introduction

Cells of the innate immune system particularly monocytes and macrophages have been recognized as pivotal players during the course of atherosclerosis, a chronic inflammatory disease (1). Accumulation of lipoproteins in the vessel wall provides the initial trigger for vascular inflammation, causing endothelial dysfunction and monocyte recruitment. Modified lipoproteins such as oxidized low-density lipoprotein (oxLDL) can activate monocyte-derived and resident macrophages by binding to scavenger receptors or pattern recognition receptors including Toll-like receptors TLR2/TLR4. Activation of macrophages by oxLDL can lead to the formation of foam cells and the secretion of proinflammatory cytokines, thereby maintaining and promoting vascular inflammation and disease progression (2, 3).

Monocyte-derived and tissue-resident macrophages are characterized by high phenotypic plasticity enabling a flexible response to the vast array of possible factors that can compromise tissue and organ function. A simplified classification discriminates between inflammatory or M1 macrophages and anti-inflammatory or M2 macrophages. However, the true macrophage phenotype is subject to a dynamic process where M1 and M2 markers and functions can coexist in a single cell (4). Depending on the trigger, the phenotypic response of innate immune cells can be quite sustained, resulting in enhanced, or reduced inflammatory responses to future insults. In this context, it is becoming increasingly clear that the functional phenotype variability of macrophages depends on the rewiring of the cellular metabolism, catering to the specific needs of the cell to carry out its required function (4). To describe a long-lasting activation or memory-like state that enables macrophages to develope an enhanced immune response to a secondary challenge, Netea et al. have coined the term “trained innate immunity” (5–7). *In vitro* and *in vivo* experiments using the Bacillus Calmette-Guerin (BCG) vaccine or the cell wall components of Candida albicans (β-glucan) demonstrated a sustained ability of monocytes and macrophages to respond with increased synthesis of chemokines and cytokines upon TLR restimulation (6, 8). While this phenotype can provide increased protection against infections, sterile inflammatory insults such as oxLDL can also induce a similar activation with potentially detrimental results in chronic inflammatory diseases such as atherosclerosis (5). Bekkering et al. reported increased expression of the inflammatory mediators TNFα, IL6, MCP-1, and MMP-9 upon restimulation with TLR2 and 4 agonists as well as increased foam cell formation 6 days after oxLDL treatment in human monocytes (9). Mechanistically, BCG, β-glucan or oxLDL treatment results in a profound metabolic and epigenetic reprogramming of the cells with increased glycolysis and enrichment of the epigenetic mark trimethylated histone H3 lysine 4 (H3K4me3) on promoter regions of induced cytokines and chemokines (6, 9, 10).

A significant shift in the redox-balance of a cell to an oxidized state can cause damage to cellular components or induce cell necrosis or apoptosis. Oxidative stress-related cell damage has long been recognized as an essential mediator in chronic inflammatory diseases including atherosclerosis (11–14). However, subtle changes in the redox state are crucial events in the regulation of many physiologic cellular functions in macrophages including transcription, differentiation and inflammatory response (11, 12, 15). Reactive oxygen species (ROS)-signaling has been demonstrated to be involved in TLR-dependent NF-κB and inflammasome activation (11, 12, 15). Furthermore, increased ROS formation leads to the activation of the transcription factor HIF1α, which is important for metabolic reprogramming during trained immunity (6, 16, 17). In this study, based on the pivotal role of the redox-balance for monocyte and macrophage function, we explored the role of ROS formation in regulating the proinflammatory priming of human monocyte derived macrophages in response to oxLDL-treatment *in vitro*.

## Materials and Methods

### PBMC and Monocyte Isolation

Human monocytes were isolated from fresh human blood leukocyte reduction chambers of platelet apheresis sets from healthy subjects recruited by the blood bank of the University Hospital Münster. The study was approved by the scientific and ethics committee of the University of Münster and conforms to the principles of the Declaration of Helsinki. Written informed consent was obtained from all donors by the blood bank and leukocyte reduction filters were provided anonymously without sharing personal and detailed information.

Monocyte isolation was then performed by differential density centrifugation over Histopaque® 1077 (Sigma, #10771) using Leucosep® tubes (50 ml, with filter, Greiner, # 227290). The cells were washed in PBS until the cell suspension in a 50 ml falcon tube looked transparent. In order to enrich monocytes, a second density centrifugation using percoll gradient was performed. Briefly, 150–200 × 10^6^ PBMCs were resuspended in RPMI-1640 (Sigma, #R8758) with 10% FBS (Sigma, #F7524), layered on top of a hyper-osmotic Percoll (GE Healthcare, #17089101) solution (46% Percoll and PBS, 10%FBS RPMI) and centrifuged for 30 min at 580 g. The interphase layer was isolated and cells were washed with PBS. Cells were purified further with MACS Pan Monocyte Isolation Kit (Miltenyi Biotec,#130-096-537) and washed once with serum-free RPMI-1640 medium before resuspension in RPMI culture medium supplemented with 10% pooled AB human serum (Sigma, H4522), 1% penicillin/streptomycin (Gibco, # 15140122) and 5 mM glucose (Sigma, G8644).

### Preparation of Oxidized LDL

LDL was isolated from the blood plasma of healthy human donors using potassium bromide (KBr) gradient after density adjustment (0.01906 and 0.06583 g/ml KBr/plasma) and two ultracentrifugation steps (2 × 24 h, 59,000 rpm at 4°C) using a type 70 Ti rotor (Beckman Coulter). KBr/plasma was layered in a Quick-Seal Ultra-Clear Tube (Beckman coulter, #344326) and after the first centrifugation the VLDL (on the top) was separated from the LDL/HDL fraction using syringes. VLDL was discarded and the solution was centrifuged for the second step. nLDL fraction (upper part) was separated from the HDL fractions and sterile filtered. NLDL was dialyzed 4 times against 1 × PBS, pH 7.4 at 4°C (1–3 h and overnight). Oxidized LDL was prepared by incubation of LDL with 20 μmol CuSO4/L for 24 h at 37°C. OxLDL was dialyzed 4 times against 1 × PBS as described before. Dialyzed oxLDL was sterile filtered and protein concentration was measured using Pierce Modified Lowry Protein Assay Kit (ThermoFisher, 23,240) following the manufactures instructions. The level of LDL peroxidation was measured using Thiobarbituric Acid Reactive Substances (TBARS) (OxiSelect TBARS Assay Kit, Cell Biolabs, #1024311). We compared high (100 μmol CuSO4/L for 3 h) and low TBAR oxLDL (20 μmol CuSO4/L for 24 h). Low TBAR oxLDL induced a higher inflammatory response (data not shown). Experiments in the manuscript were performed with low TBAR LDL. Possible endotoxin contamination was checked with ToxinSensor chromogenic LAL endotoxin assay kit (GenScript, ABIN491527) based on the modified Limulus Amebocyte Lysate (LAL) method. All nLDL or oxLDL which was used in the experiments had endotoxin levels lower than 0.01 EU/ml.

### Monocyte Priming Experiments

Monocytes were primed by culturing 40,000 cells/well in a 96-well plate (Greiner Bio*-*One™) with 20 μg/ml of oxLDL for 24 h in RPMI supplemented with 10% pooled human AB serum, 5 mM glucose and 1% Penicillin/Streptomycin. In experiments in which inhibitors were used, cells were pre-incubated for 1 h with 100 nm of Torin1 (Cayman, #10997), 40 μM Mito-TEMPO (Sigma, #SML0737), 0.5 μM of Diphenyleneiodonium (DPI) (Sigma, #D2926), 25 μM VAS2870 (Sigma, #SML0273) or 200 μM of tert-Butyl hydroperoxide solution (Sigma, # 416665) prior to oxLDL treatment. The medium was changed after 24 h and cells were let to rest for 5 days or as indicated. 50% of the medium was refreshed on day 3. On day 6 medium was changed and cells were re-stimulated with either 200 μL RPMI or 5 μg/ml of Pam3Cys (EMC mirocollection, #L2000). The plates were incubated in a 5% CO_2_ incubator maintained at 37°C. After 24 h supernatants were collected and stored at −20°C until used for cytokine assay. Stock solution for Pam3cys and MitoTEMPO were prepared in water and Torin1, DPI and VAS2870 were dissolved in DMSO according to the manufacturer's recommendation. DMSO control was always included when there was a compound dissolved in DMSO. All working solutions were prepared in culture media.

### Apoptosis Assay

Monocytes were treated with 10, 20, and 50 μg/ml oxLDL in supplemented culture medium for 24 h. Cells were collected and stained with FITC conjugated Annexin V (BioLegend, #640914) and propidium iodide (PI) (Sigma, #P4170), following the manufacturer's instructions. Briefly, cells were collected, centrifuged, washed once in PBS + 0.5%BSA and stained with FITC Annexin V for 15 min. For staining of necrotic cells, PI was added briefly before analysis. Cells were analyzed by FACS (Guava easyCyte, Millipore) and the percentage of necrotic and apoptotic cells were calculated.

### Cytokine Measurements

The levels of the proinflammatory cytokines in supernatants were measured using DuoSet ELISA kits for human TNFα (R&D, #DY210) and human IL-6 (R&D, #DY206) following the instructions of the manufacturer. The absorbance was quantified in a *Multimode Plate Reader Victor X3*, P Perkin Elmer (USA) at 450 nm. Concentrations were calculated by four parameters logistic regression.

### Gene Silencing by siRNA

Cells were seeded on either 24- or 96-well cell culture treated plates at a concentration of 500,000 and 60,000 cells/well, respectively. The cells were transfected with final concentrations of 60 nM small interfering RNA for Raptor (Santa Cruz, #sc-44069) and Rictor (Santa Cruz, #sc-61478) for 24 h using Viromer technology according to the manufacturer's protocol (Viromer Green, Lipocalyx). Scramble siRNA (Santa Cruz, #sc-37007) was used as a control for the experiments. Knockdown of Raptor and Rictor were confirmed by qPCR analysis 24 h post transfection. For further analysis, 24 h after transfection cells were either left untreated or treated with 20 μg/ml oxLDL as described earlier in monocyte priming protocol. The level of mitochondrial ROS (MitoROS), cytosolic ROS (CytoROS), p-mTOR, Hif1α accumulation, IL-6 and TNFα concentration were analyzed as described in the respective paragraphs of the Materials and Methods section.

### Intracellular Reactive Oxygen Species Analysis

The level of cytosolic and mitochondrial ROS were quantified 24 h after oxLDL priming by FACS using CellROX green (Invitrogen, # C10444) and MitoSOX red (Invitrogen, #M36008) dyes, respectively. Cells were washed once with PBS and were stained by adding 50 μl of 5 μM CellROX or 2.5 μM MitoSOX in Hanks' balanced salt solution and subsequently incubated at 37°C for 30 min in dark conditions. Cells were then washed twice with PBS and held on ice until analyzed by flow cytometry (Guava easyCyte, Millipore) or microscopy using 20x magnification (Leica Microsystems). Data acquisition and analysis was performed using the InCyte software and ImageJ software for FACS and microscopic images, respectively.

### Western Blotting

For Western blotting, 10 × 10^6^ human monocytes were seeded in 10 cm Petri dishes in 10 ml of RPMI medium containing 10% of human serum. The cells were incubated with oxLDL 20 μg/ml or treated with inhibitors according to the protocol mentioned above. After the resting time on day 6, the cells were harvested for western blotting using RIPA buffer containing 1% phosphatase (Thermo Scientific, #78426) and 1% protease (Thermo Scientific, #87786) inhibitors. Bio-Rad DC Protein Assay was used to perform protein quantification and equal amounts of proteins were loaded on SDS-PAGE. Proteins were transferred on a PVDF membrane (GE Healthcare, #10600023) and blocked in 5% milk (w/v) in Tris-buffered saline supplemented with Tween 20 (TBS-T). Membranes were incubated with a primary antibody [rabbit anti-Phospho-mTOR (Ser2448) (Cell Signaling, #2971S), mouse anti-mTOR (6H9B10) (Biolegend, #659201), rabbit anti-Phospho-p70 S6 Kinase (Thr389) (Invitrogen, #710095); mouse anti-Vinculin (7F9) (Santa Cruz, #sc-73614)], in TBS-T overnight at 4°C, followed by washing with TBST and incubation with a secondary antibody (Goat anti-rb IgG-HRP (Santa Cruz, #sc-2004) or Goat anti-mouse IgG-HRP (Santa Cruz, #sc-2005) in TBS-T for 1 h. The blots were washed as above and developed using Pierce western blotting substrate (Thermo. Scientific, #32106) and images were captured using an Amersham Imager 600 (GE Healthcare). The intensity of bands was quantified and normalized using ImageJ software.

### RNA Isolation and qPCR

For real-time qPCR monocytes were treated with oxLDL as described above and lysed for mRNA isolation at the indicated time points. For analyzing expression of cytokines/chemokines the cells were stimulated with 5 μg/ml of Pam3Cys for 6 h before lysing. Total RNA purification was performed using NucleoSpin RNA-isolation kit (Macherey-Nagel) and reverse-transcribed using the RevertAid First Strand cDNA Synthesis Kit (Thermo Scientific). Expression of *NOX2, NOX4, LDH, PDK1*, and *PFKFB3* was analyzed on day 3 and expression of *IL6, TNF*α, *MCP-1, Glut1 and LDH* were analyzed on day 6 using iTaq™ Universal SYBR® Green supermix (Bio-Rad, #172-5124). Samples were analyzed following a quantitative method with efficiency correction, and TFIIB was used as a housekeeping gene. Primer sequences are available on request.

### Lactate Assay

Intracellular Lactate was measured using a colorimetric L-Lactate assay kit according to the manufacturer's instructions (Abcam, #ab65330). Cells were cultured in a 6 well plate and treated with oxLDL for 24 h. On day 6 the cells were washed with ice cold PBS and scraped from the plate and lysed with assay buffer. To eliminate endogenous LDH, cell lysate was deproteinized by spinning through a 10 kD Spin column (Abcam, #ab93349). Absorbance was measured with a CLARIOstar Microplate Reader at 570 nm and the level of lactate was calculated.

### NADP/NADPH Assay

NADP/NADPH levels were measured using a colorimetric NADP/NADPH assay kit (Abcam, #ab65349) according to the manufacturer's instructions. OxLDL treated cells were lysed on day 6 in an assay buffer provided in the kit. The lysates were deproteinized by passing through a 10 kD Spin column (Abcam, #ab93349). The assay was performed in a 96-well plate and absorbance was measured with a Multimode Plate Reader Victor X3, P Perkin Elmer (USA) at 450 nm.

### Intracellular Staining and FACS

On day 1, day 3, and day 6 primed cells were harvested using ice cold PBS containing 5 mM EDTA. Cells were washed with PBS and staining was performed for 30 min on ice with surface markers for CD80-APC (Biolegend, #305220), CD86-VioBright 515 (Miltenyi Biotec, #130-116-165), CD163-APC-Vio770 (Miltenyi Biotec, #130-112-131) and CD206 (Biolegend, #321110) and following manufacturer's instructions. Intracellular staining of p-mTOR and HIF1α was performed after 4% formaldehyde fixation. Cells were washed once with permeabilization buffer (Biolegend, #421002) and stained with PE-Cyanine7 anti-human p-mTOR (Ser2448) (eBioscience, #25-9718-41) or PE anti-human HIF1α Antibody (Biolegend, #359704) antibodies for 20 min in the dark at room temperature. PE mouse IgG2b κ (Biolegend) and PE-Cyanine7 mouse IgG2a κ (eBioscience™) were used as isotype control for intracellular staining. After staining the cells were washed with PBS and FACS analysis was performed using Guava easyCyte (Millipore).

### Statistical Analysis

The differences among experimental groups were evaluated with the Mann–Whitney test using Graph pad prism for Windows. A two-sample *t*-test was used to compare the mean levels in case there were only two groups to be compared. A *p* < 0.05 was considered to be statistically significant.

## Results

### OxLDL Priming of Human Monocytes Depends on mTOR Activation

**Figure 1:**
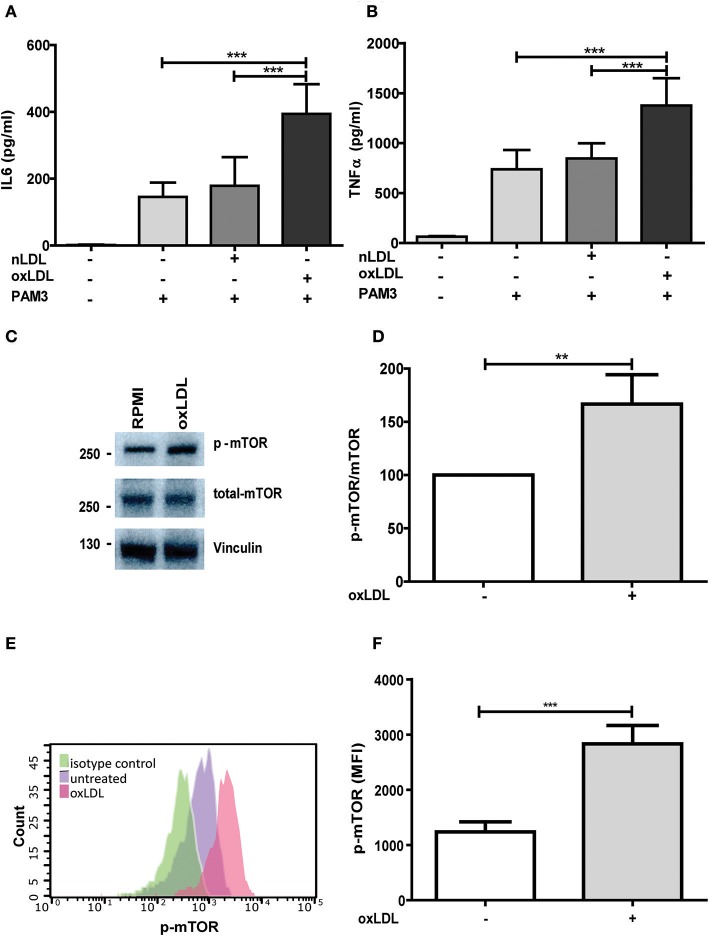
OxLDL priming induces inflammatory response and activation of mTOR. Monocytes were treated 20 μg/ml oxLDL or vehicle for 24 h, kept for 6 days in complete medium and restimulated with 5 μg/ml Pam3cys for 24 h. IL-6 **(A)** and TNFα **(B)** were measured in the supernatant. **(C)** Monocytes were treated as described above and lysed on day 6. Whole-cell extracts were subjected to western blot analysis for p-mTOR and mTOR on day 6. **(D)** Densitometry of p-mTOR/mTOR western blot analysis. **(E,F)** oxLDL primed cells and untreated cells were stained with PE-Cyanine7 anti-human p-mTOR on day 6 and analyzed by FACS. The MFI (mean fluorescence intensity) of p-mTOR was compared between untreated cells and oxLDL primed cells. Graphs represent mean values ± SD of at least 6 individuals in at least 3 different experiments. ^**^P < 0.01 and ^***^P < 0.001.

The concept of trained innate immunity postulated by Netea et al. describes an activated macrophage phenotype that enables an enhanced inflammatory cytokine production in response to a secondary challenge (5, 18). BCG, β-glucan, or oxLDL have been described as inducers of this activated phenotype. For our experiments, we applied the cell culture model established by Bekkering et al. using oxLDL to induce the activated proinflammatory phenotype in human monocyte derived macrophages (9). Human monocytes were treated with 20 μg/ml oxLDL, a dose that did not induce apoptosis or necrosis (Figure S1), and rested for 5 days to induce macrophage differentiation. oxLDL treated monocyte derived macrophages displayed a significant induction of M1 (CD 80, CD 86) markers without a corresponding decrease in M2 markers (CD 163, CD 206) compared to unprimed macrophages (Figure S2). Furthermore, oxLDL treatment induced increased secretion of IL6 and TNFα (Figures 1A,B) as well as increased mRNA expression of IL6, TNFα, and MCP-1 (Figure S3) upon restimulation with the TLR2-agonist Pam3cys. These observations are in line with previous reports by Bekkering et al. reporting a similar oxLDL-induced proinflammatory macrophage phenotype that displayed both M1 and M2 phenotypic markers (9).

**Figure 2:**
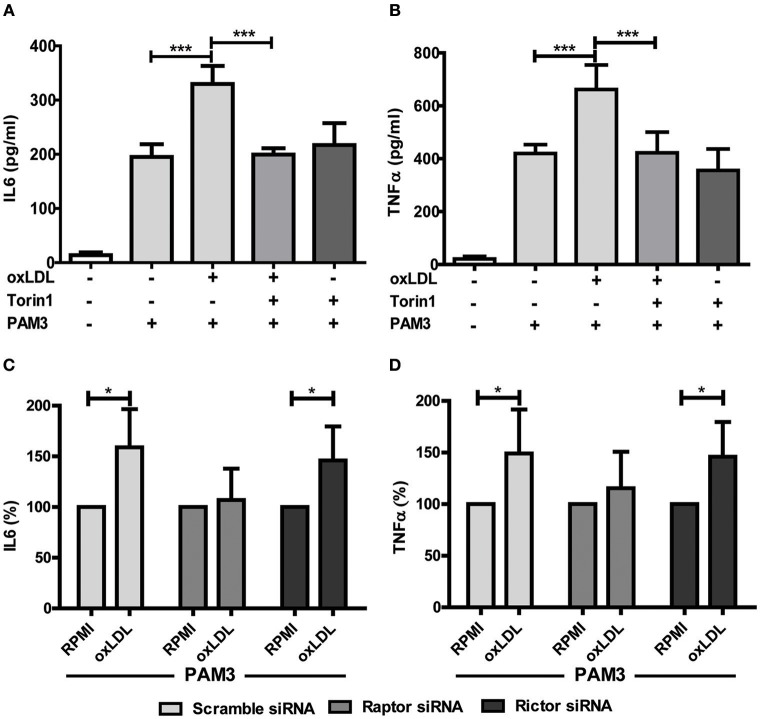
mTOR inhibition blocks oxLDL-induced priming in monocytes. Cells were pretreated with Torin1 (100 nM) for an hour and stimulated with oxLDL (20 μg/ml) or vehicle. On day 6 cells were restimulated with 5 μg/ml Pam3cys for 24 h and IL6 **(A)** and TNFα **(B)** production was measured in the supernatant using ELISA. **(C,D)** Monocytes were transfected with a final concentration of 60 nm Raptor or Rictor siRNA. Cells were treated with 20 μg/ml oxLDL or vehicle for 24 h. On day 6 cells were stimulated with Pam3cys for 24 h. IL6 **(C)** and TNFα **(D)** were measured in the supernatant. *P < 0.05 and ***P < 0.001.

**Figure 3:**
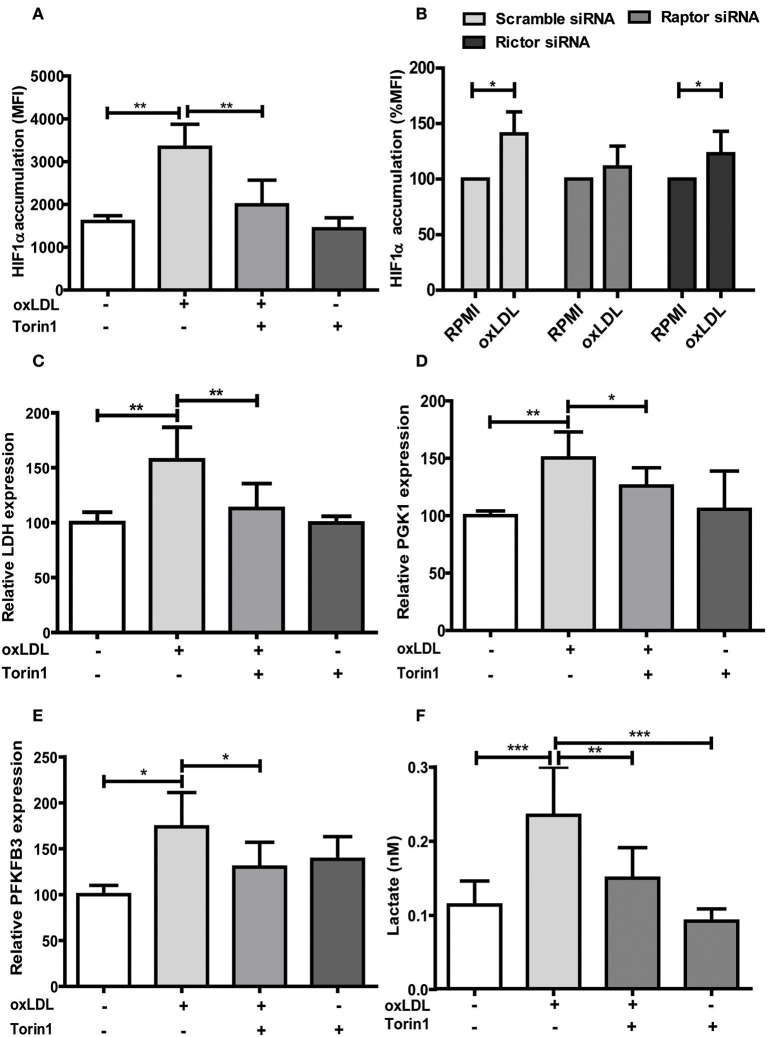
mTOR inhibition alters HIF1α accumulation, HIF1α target gene expression and lactate production. **(A)** Cells were treated with 100 nM Torin1 or vehicle an hour before treatment with 20 μg/ml oxLDL or vehicle. The cells were harvested on day 6, stained with PE anti-human HIF1α Antibody and analyzed by FACS. The MFI (mean fluorescence intensity) was compared. **(B)** Monocytes were transfected with a final concentration of 60 nm Raptor or Rictor siRNA. Cells were then treated with 20 μg/ml oxLDL or vehicle for 24 h. HIF1α accumulation was measured as described above on day 6. **(C–E)** Cells were treated with Torin1 and oxLDL as described above. Cells were then harvested on day 3 and mRNA expression of LDH **(C)**, PGK1 **(D)** and PFKFB3 **(E)** was measured using real-time qPCR. **(F)** Cells were treated with Torin1 and oxLDL as described above. On day 6 cells were harvested and lactate concentration was measured using a colorimetric assay. Graphs represent mean values ± SD of 6 biological replicates in at least 3 different experiments. *P < 0.05, **P < 0.01 and ***P < 0.001.

Treatment with non-oxidized LDL had no effect (Figures 1A,B) (9). Next, we analyzed the role of the mTOR-HIF1α-axis in oxLDL-induced inflammatory priming, which has been shown to be required for the metabolic reprogramming necessary for the trained innate immunity phenotype induced by BCG or β-glucan treatment (6, 19). Activation of the mTOR target gene HIF1α controls the upregulation of the glycolytic capacity to allow ATP production under hypoxic conditions but can increase aerobic glycolysis and subsequent pyruvate and lactate production in myeloid cells as well. Inhibition of HIF1α impairs the inflammatory response of macrophages and blocks the emergence of a trained innate immunity phenotype in BCG and β-glucan treated cells (6, 20, 21). Similar to β-glucan or BCG, oxLDL training also activated mTOR signaling as demonstrated by increased phosphorylation of mTOR and p70S6K (Figures 1C,D; Figures S4, S5). As depicted in Figure S4 and Figures 1E,F, mTOR phosphorylation was still detectable after 3 and 6 days, demonstrating a sustained activation of this important metabolic regulator. Illustrating the functional relevance of mTOR-activation, pharmacologic inhibition of mTOR using the mTOR-inhibitor Torin1 (Figure S6) could block the emergence of the trained phenotype (Figures 2A,B). mTOR is the catalytic subunit of two distinct protein complexes, mTORC1 and mTORC2. While mTORC1 controls macrophage metabolism, activation and polarization, much less is known about the functions of mTORC2 (22, 23). To analyze the contribution of mTORC1 and mTORC2 to the observed phenotype we performed siRNA experiments against the adapter proteins Raptor and Rictor which are specific to the respective complexes (Figure S7). As shown in Figures 2C,D knockdown of Raptor inhibited the trained immunity phenotype while knockdown of Rictor had no significant effect. As mTOR-dependent HIF1α accumulation is a prerequisite for the profound metabolic reprogramming during trained innate immunity (6, 10), we also analyzed HIF1α protein levels, HIF1α target genes and lactate production following oxLDL priming and mTOR inhibition. As demonstrated in Figure 3 oxLDL treatment increased HIF1α levels (Figures 3A,B; Figure S8), mRNA expression of HIF1α target genes (Figures 3C–E) and lactate production (Figure 3F), indicating increased glycolysis, while Torin1 blocked these effects (Figures 3A,C–F, Figure S8). siRNA mediated knockdown of Raptor (Figure 3B) was also able to inhibit the increase in HIF1α levels.

### OxLDL Training Induces ROS Formation in a mTOR-Dependent Manner

**Figure 4:**
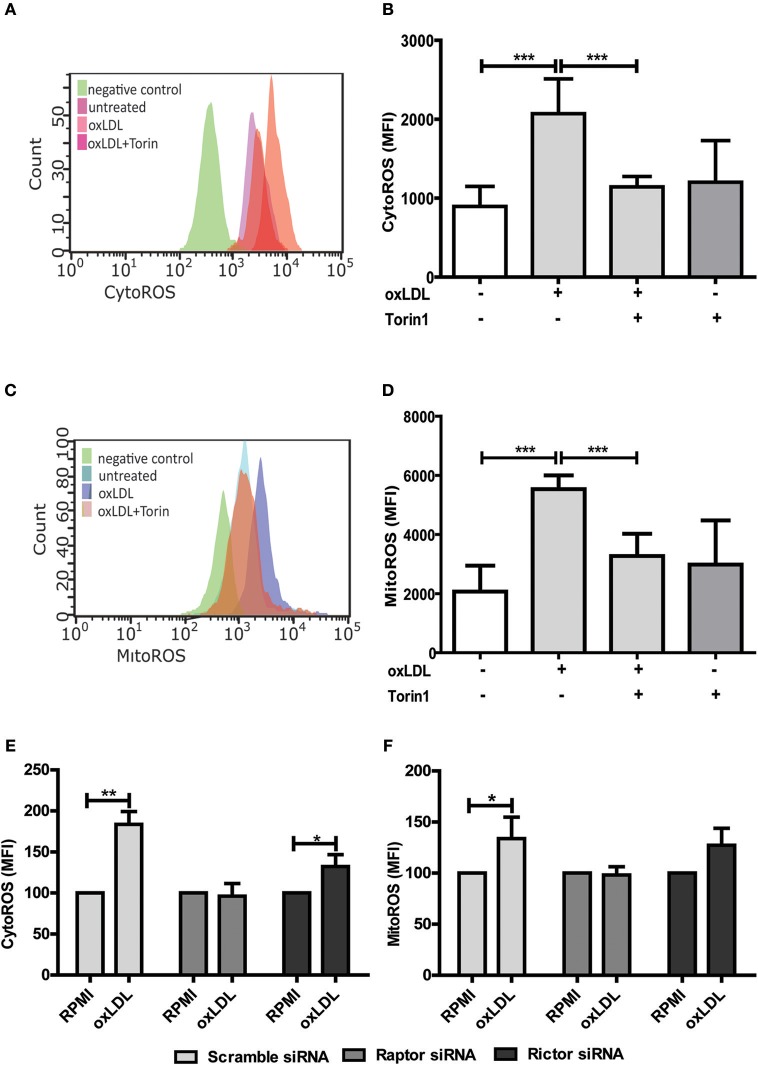
mTOR inhibition reduces oxLDL induced ROS generation in monocytes. Cells were primed with 20 μg/ml oxLDL in the presence or absence of 100 nM Torin1 for 24 h. Primed monocytes were stained for cytosolic ROS (CytoROS) using 5 μM CellROX green or mitochondrial ROS (MitoROS) using 2.5 μM MitoSOX red and analyzed by FACS. Histogram overlays and columns show intensity of CellROX **(A,B)** and MitoSOX **(C,D)**. Monocytes were transfected with a final concentration of 60 nm Raptor or Rictor siRNA. Cells were then treated with 20 μg/ml oxLDL or vehicle for 24 h and CytoROS **(E)** and MitoROS **(F)** were measured by FACS analysis. Graphs represent mean values ± SD of at least 6 individuals in at least 3 different experiments. *P < 0.05, **P < 0.01 and ***P < 0.001.

As mentioned above, ROS constitute an important regulator of monocyte and macrophage functions including transcription, differentiation and inflammatory response (11, 12, 15). As depicted in Figure S9, oxLDL doses used in our cell culture model cause a significant increase in cytosolic and mitochondrial ROS formation. Furthermore, oxLDL-induced ROS formation was mediated through mTOR as pharmacologic inhibition of mTOR was sufficient to block the increase in ROS level (Figures 4A–D). siRNA mediated knockdown of the mTORC1 and mTORC2 adapter proteins Raptor and Rictor respectively demonstrated a privileged role for mTORC1 in oxLDL-induced ROS formation (Figures 4E,F).

### Antioxidant Treatment Blocks oxLDL Training

**Figure 5:**
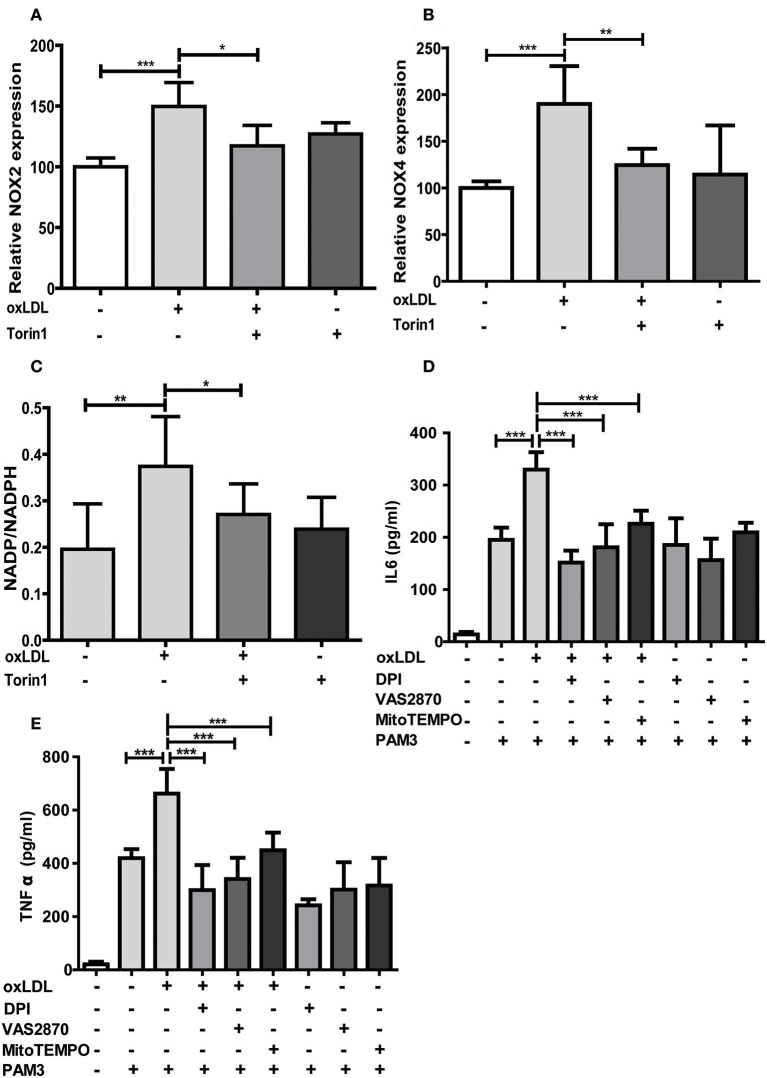
mTOR inhibition reduces NOX expression and antioxidants block oxLDL priming. Cells were treated with 100 nM Torin1 or vehicle an hour before treatment with 20 μg/ml oxLDL or vehicle. On day 3 cells were harvested and mRNA expression of NOX2 **(A)** and NOX4 **(B)** was analyzed using real-time qPCR. **(C)** Cells were treated as described above. Concentrations of NADP and NADPH were measured using a colorimetric assay kit on day 6. Ratio of NADP/NADPH is shown. **(D,E)** Monocytes were pre-incubated for 1 h with 0.5 μM Diphenyleneiodonium (DPI), 25 μM VAS2870, 40 μM Mito-TEMPO or vehicle before treating with oxLDL or vehicle for 24 h. On day 6 cells were stimulated with Pam3cys and IL6 **(D)** and TNFα **(E)** were measured in the supernatant using ELISA. Graphs represent mean values ± SD of at least 6 individuals in at least 3 different experiments. *P < 0.05, **P < 0.01 and ***P < 0.001.

As oxLDL treatment of monocytes is known to induce ROS formation through increased expression and activity of NADPH oxidases (Nox), particularly Nox2 and Nox4 (24, 25), we investigated if this effect is also regulated through mTOR and relevant for oxLDL inflammatory priming. As depicted in Figures 5A,B mTOR inhibition could block oxLDL-induced expression of Nox2 and Nox4. The cellular NADP/NADPH ratio was also increased following oxLDL treatment, suggesting enhanced Nox activity (26), and mTOR inhibition could block this increase (Figure 5C). Next, we investigated if direct inhibition of ROS by antioxidant treatment could block the induction of trained innate immunity. To reduce ROS levels we used DPI, a Nox and mitochondrial ROS formation inhibitor (27, 28), VAS2870, a pan-Nox inhibitor (29), and MitoTEMPO, a mitochondria-targeted antioxidant with superoxide and alkyl radical scavenging properties (30). As expected, DPI significantly reduced cytosolic and mitochondrial ROS levels while VAS2870 treatment decreased cytosolic and MitoTEMPO mitochondrial ROS levels (Figure S10). All 3 compounds inhibited the proinflammatory priming in response to oxLDL. However, DPI and VAS2870 exhibited a stronger effect than MitoTEMPO indicating that cytosolic ROS could be more important than mitochondrial ROS (Figures 5D,E). Furthermore, we analyzed the effect of artificially increased oxidative stress on trained immunity. Monocytes were treated with tert-butyl peroxide together with oxLDL. As shown in Figure S11 this treatment resulted in synergistic induction of IL6 secretion upon Pam3cys restimulation.

**Figure 6:**
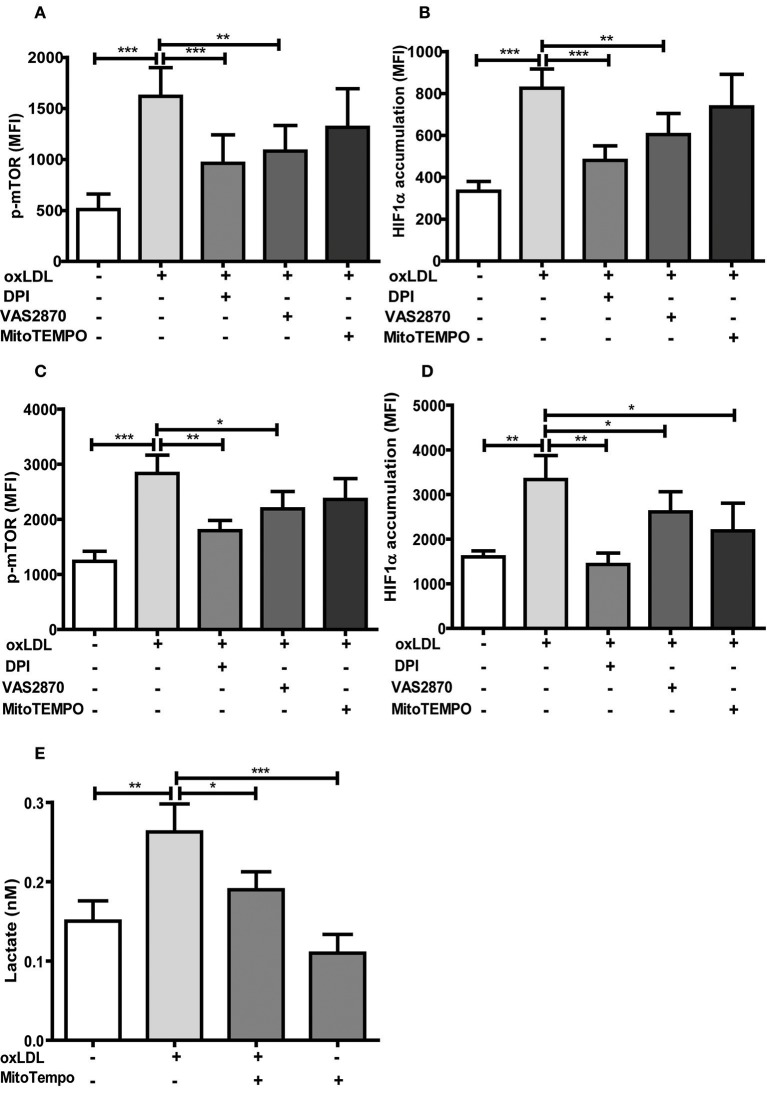
Antioxidants inhibit activation of the mTOR-HIF1α-axis. Monocytes were pre-incubated for 1 h with 0.5 μM Diphenyleneiodonium (DPI), 25 μM VAS2870 or 40 μM Mito-TEMPO or vehicle and treated with oxLDL or vehicle for 24 h. Phosphorylation of mTOR and HIF1α accumulation was assessed by staining with PE-Cyanine7 anti-human p-mTOR and PE anti-human HIF1α Antibody and analyzed by FACS on day 1 **(A,B)** or day 6 **(C,D)**. The MFI (mean fluorescence intensity) was compared between experimental groups. **(E)** Cells were pre-incubated for 1 h with 40 μM Mito-TEMPO or vehicle and treated with oxLDL or vehicle for 24 h. Lactate concentration was measured on day 6 cells using a colorimetric assay kit. Graphs represent mean values ± SD of at least 6 individuals in at least 3 different experiments. *P < 0.05, **P < 0.01 and ***P < 0.001.

DPI and VAS2870 treatment also inhibited acute and sustained oxLDL-induced mTOR phosphorylation and HIF1α accumulation (Figures 6A–D). Interestingly, MitoTEMPO did not significantly reduce mTOR activation and HIF1α levels at 24 h and mTOR activation on 6 days but did reduce HIF1α levels and HIF1α target gene expression at day 6 (Figures 6A–D; Figure S12) illustrating that both cytosolic and mitochondrial ROS compartments are important to induce the persistent proinflammatory phenotype. In line with this late reduction of HIF1α levels by MitoTEMPO, we could also show that mitochondrial ROS inhibition by MitoTEMPO blocked increased lactate production as a marker of glycolysis (Figure 6E). These experiments demonstrate that mTOR-dependent ROS formation is necessary for the emergence of a trained immunity phenotype.

## Discussion

A “trained” macrophage phenotype has been suggested to contribute to chronic vascular inflammation, a fundamental mechanism of atherosclerosis formation (5). In the present study, we report a pivotal role of oxidative stress as a novel mechanism regulating oxLDL induced inflammatory priming in human monocyte derived macrophages.

**Figure 7:**
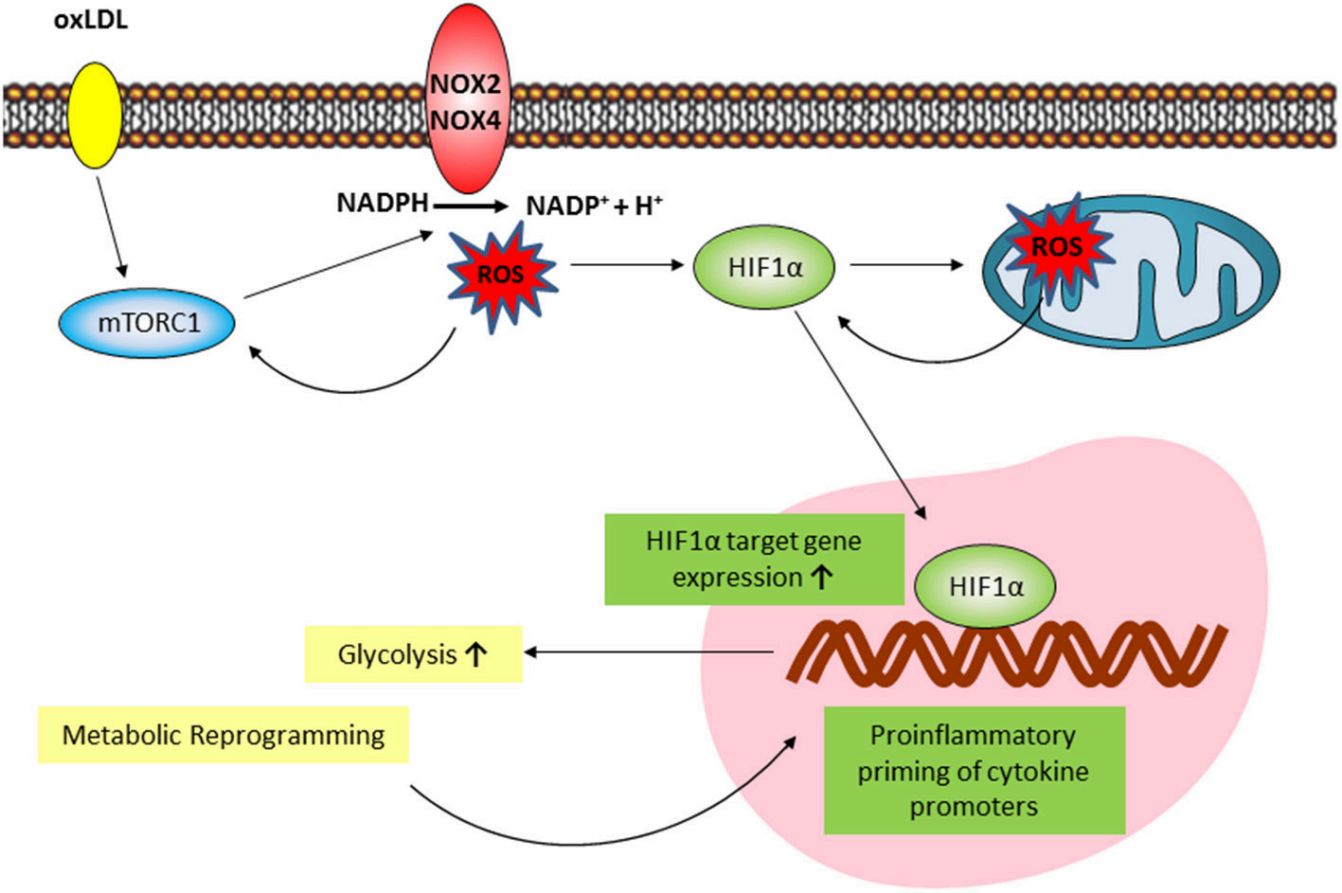
Proposed model of the mTOR/HIF1α/ROS-dependent regulation of oxLDL inflammatory priming. oxLDL activation of mTORC1 induces a NOX-dependent increase in cytosolic ROS formation. Cytosolic ROS increases mTORC1 activation through a positive feedback loop and is necessary for HIF1α accumulation. Downstream increased HIF1α target gene expression activates glycolysis and the global metabolic reprogramming necessary for inflammatory priming. mTOR/HIF1α-dependent mitochondrial ROS formation is necessary for long-term HIF1α protein stabilization and HIF1α target gene expression.

Macrophage phenotypic plasticity is a dynamic process enabling flexible immune responses to different environmental stimuli. This phenotypic plasticity is complemented by the ability of innate immune cells to acquire a more stable “memory-like” phenotype following specific priming. This innate immune memory enables the cells to answer an unrelated future challenge with a modulated response that can be enhanced, reduced or both (31). In general, macrophage activation and phenotypic modulation are accompanied by a significant and variable metabolic reprogramming that appears to be specific to the initiating stimulus. In case of an innate immune memory, the ability to conserve a modulated response following a priming stimulus requires a persistent metabolic rewiring and epigenetic reprogramming of the cell (32). The trained innate immunity phenotype postulated by Netea et al. describes an enhanced secondary response to a certain stimulus and involves increased aerobic glycolysis and positioning of activating histone modifications on cytokine promoters as basic characteristics (6). However, the metabolic and cellular signaling pathways involved in this trained innate immunity can differ depending on the initial stimulus (10). Although known inducers of trained innate immunity such as β-glucan, oxLDL, BCG, mevalonate, or fumarate all lead to an enhanced inflammatory response, metabolic, epigenetic and gene expression patterns only partly overlap (10, 18, 19, 33, 34). This observation reflects the described ability of macrophages to variably adjust their phenotypic response to the perceived environmental cues. Previous reports on the effect of oxLDL treatment on monocyte to macrophage differentiation have been conflicting, with reports claiming anti- as well as pro-inflammatory macrophage phenotypes following oxLDL incubation of monocytes (35). Our data clearly support the findings by Bekkering et al. showing that low dose oxLDL treatment of primary human monocytes induces a proinflammatory phenotype following differentiation into macrophages with enhanced cytokine secretion in response to TLR-agonist restimulation. Bekkering et al. have also shown that similar to β-glucan, oxLDL priming involves TLR-signaling, the PI3K-AKT-pathway, increased glycolysis, inhibition by fluvastatin treatment and increased histone H3K4 methylation on cytokine promoters (9, 10, 33). In our experiments we observed some additional consistencies between β-glucan and oxLDL priming. oxLDL treatment activated mTOR-signaling and HIF1α expression which are crucial to β-glucan induced metabolic reprogramming and immune memory (6, 10), while pharmacologic mTOR-inhibition or siRNA mediated knockdown of the mTORC1 subunit Raptor were able to block the enhanced inflammatory response. In line with a “memory-like” reprogramming of the cells, we could show that the mTOR-HIF1α-axis was still activated 6 days after oxLDL treatment and throughout the monocyte to macrophage differentiation process. Although both β-glucan and oxLDL priming induces a proinflammatory phenotype in macrophages in an mTOR-dependent manner, oxLDL priming induces ROS production while β-glucan training does not (33). Furthermore, previous research has also shown that β-glucan can have antioxidant effects *in vitro* and *in vivo* (36). In fact, our experiments demonstrated that ROS formation appears to be a pivotal component of the cellular signaling cascade that shapes the oxLDL-induced memory (33) as modulation of the cellular redox-balance by antioxidant treatment or tert-butyl peroxide could block or superinduce the inflammatory response, respectively. This observation further highlights the trigger dependent differences in the macrophage phenotype regulation and suggests ROS production following oxLDL priming to be a crucial factor that controls the differential regulation of trained innate immunity phenotypes. OxLDL has been shown to induce mTOR-phosphorylation in human monocytes (37), ROS formation (24) and HIF1α accumulation via redox-dependent mechanisms (16, 38). Our data extend these observations in the context of monocyte to macrophage differentiation and demonstrate that cytosolic and mitochondrial ROS formation are crucial regulators of the mTOR-HIF1α-axis ultimately controlling the metabolic reprogramming and the emergence of a trained proinflammatory macrophage phenotype in response to oxLDL priming (Figure 7). mTOR activation induced Nox activity and cytosolic ROS formation, while inhibition of cytosolic ROS through DPI or Nox-inhibition could also block mTOR-phosphorylation suggesting a positive feedback loop between mTOR activation and cytosolic ROS formation. mTOR inhibition could also block mitochondrial ROS formation and scavenging of mitochondrial ROS could inhibit increased lactate production and inflammatory training. Our observation that mitochondrial ROS scavenging did not block mTOR activation or HIF1α-accumulation at an early time point (24 h) but significantly reduced HIF1α-levels at day 6, suggests that mitochondrial ROS formation occurs initially downstream of the mTOR-cytoROS-HIF1α feedback loop but appears to be a crucial factor that controls the long-term activation of the mTOR-HIF1α-axis. The regulation of HIF1α stabilization, transcription and transactivation by mitochondrial ROS in various cell culture models has been known for many years (39). However, as the precise mechanisms governing the regulation of HIF1α by ROS have been under debate for a long time, they are beyond the scope of this manuscript. Our data warrant additional research to elucidate the molecular details of the ROS-dependent regulation of mTOR and HIF1α in this model.

In summary, we demonstrate that mTOR dependent ROS production controls oxLDL-induced trained innate immunity in human monocytes with different roles for cytosolic and mitochondrial ROS. Further research is necessary to elucidate the detailed mechanisms of mTOR-dependent cytosolic and mitochondrial ROS formation in trained innate immunity and their impact on the metabolic and inflammatory phenotype. Pharmacologic modulation of these pathways might provide a promising approach to modulate inflammation during atherosclerosis development.

## Author Contributions

YS designed and performed experiments, and contributed to the writing of the manuscript, SMML performed experiments, LS performed experiments, RG performed experiments, FK assisted in designing and performing experiments, DB assisted in designing and performing experiments, JW designed experiments, assisted in drafting the manuscript, HF designed the study, drafted the manuscript.

### Conflict of Interest Statement

The authors declare that the research was conducted in the absence of any commercial or financial relationships that could be construed as a potential conflict of interest.

## Abbreviations

oxLDL: oxidized low-density lipoprotein
ROS: reactive oxygen species
TLR2: toll-like receptor 2
mTOR: mechanistic target of rapamycin
mTORC: mechanistic target of rapamycin complex
Nox: NADPH oxidase
HIF1α: hypoxia-inducible factor 1α
CytoROS: cytosolic reactive oxygen species
MitoROS: mitochondrial reactive oxygen species
BGC: Bacillus Calmette-Guerin vaccine
TNFα: tumor necrosis factor alpha
IL6: interleukin 6
MCP-1: Monocyte chemoattractant protein 1
MMP-9: Matrix metallopeptidase 9
H3K4me3: trimethylated histone H3 lysine 4
NF-κB: nuclear factor kappa-light-chain-enhancer of activated B cells
PBS: phosphate buffered saline
PBMCs: Peripheral Blood Mononuclear Cell
RPMI: Roswell Park Memorial Institute medium
FBS: fetal bovine serum
nLDL: native low-density lipoprotein
KBr: potassium bromide
TBAR: Thiobarbituric acid reactive substances
DPI: Diphenyleneiodonium
PAM3: Pam3Cys
PI: propidium iodide
FITC: Fluorescein isothiocyanate
BSA: bovine serum albumin
RIPA: Radioimmunoprecipitation assay buffer
SDS-PAGE: sodium dodecyl sulfate polyacrylamide gel electrophoresis
HRP: horseradish peroxidase
Glut1: Glucose transporter 1
LDH: lactate dehydrogenase
PDK1: Pyruvate dehydrogenase kinase 1
PFKFB3: 6-phosphofructo-2-kinase/fructose-2, 6-biphosphatase 3
TFIIB: Transcription factor II B
ATP: Adenosine triphosphate.
