# Supplementary material for: mTOR-Dependent Oxidative Stress Regulates oxLDL-Induced Trained Innate Immunity in Human Monocytes
Source: Front Immunol. 2019 Jan 22;9:3155. doi: 10.3389/fimmu.2018.03155 (PMC6350618; doi:10.3389/fimmu.2018.03155)
Supplement: Supplementary file 1 [file Presentation_1.pdf]

Supplementary material

mTOR-Dependent Oxidative Stress Regulates oxLDL-Induced Trained Innate Immunity in Human Monocytes

Yahya Sohrabi, Sina M. M. Lagache, Lucia Schnack, Rinesh Godfrey, Florian Kahles, Dennis Bruemmer, Johannes Waltenberger, Hannes M. Findeisen*

Correspondence: Hannes M. Findeisen: hannes.findeisen@ukmuenster.de

Supplementary Figures

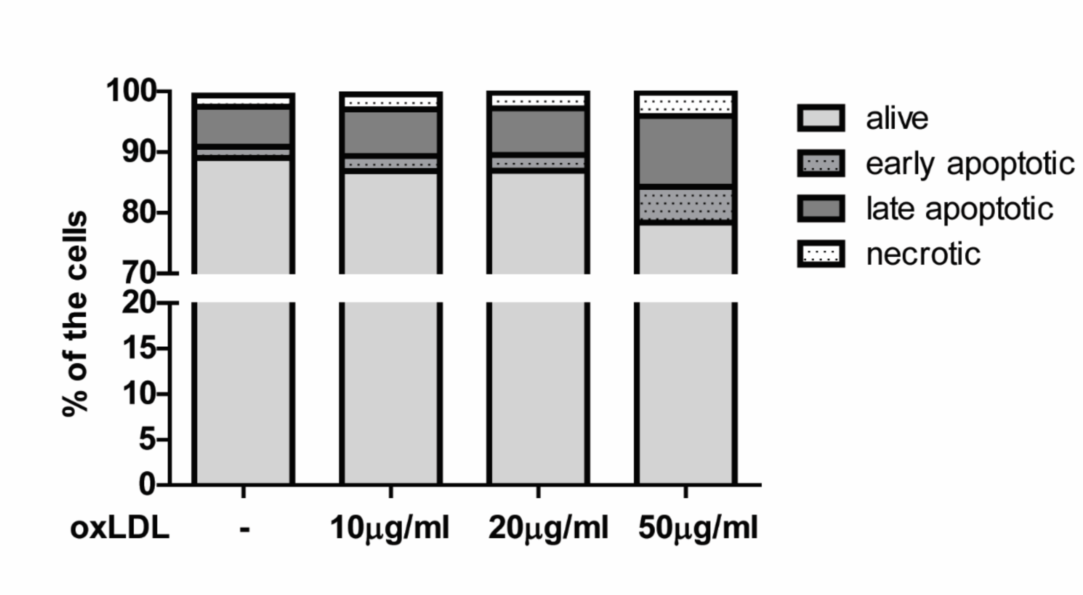

Figure S1.
Low dose oxLDL treatment does not induce apoptosis. Monocytes were treated with 10, 20 and 50µg/ml of oxLDL or vehicle for 24h. Cells were stained with Annexin V and PI for apoptosis assay and were analyzed by FACS.

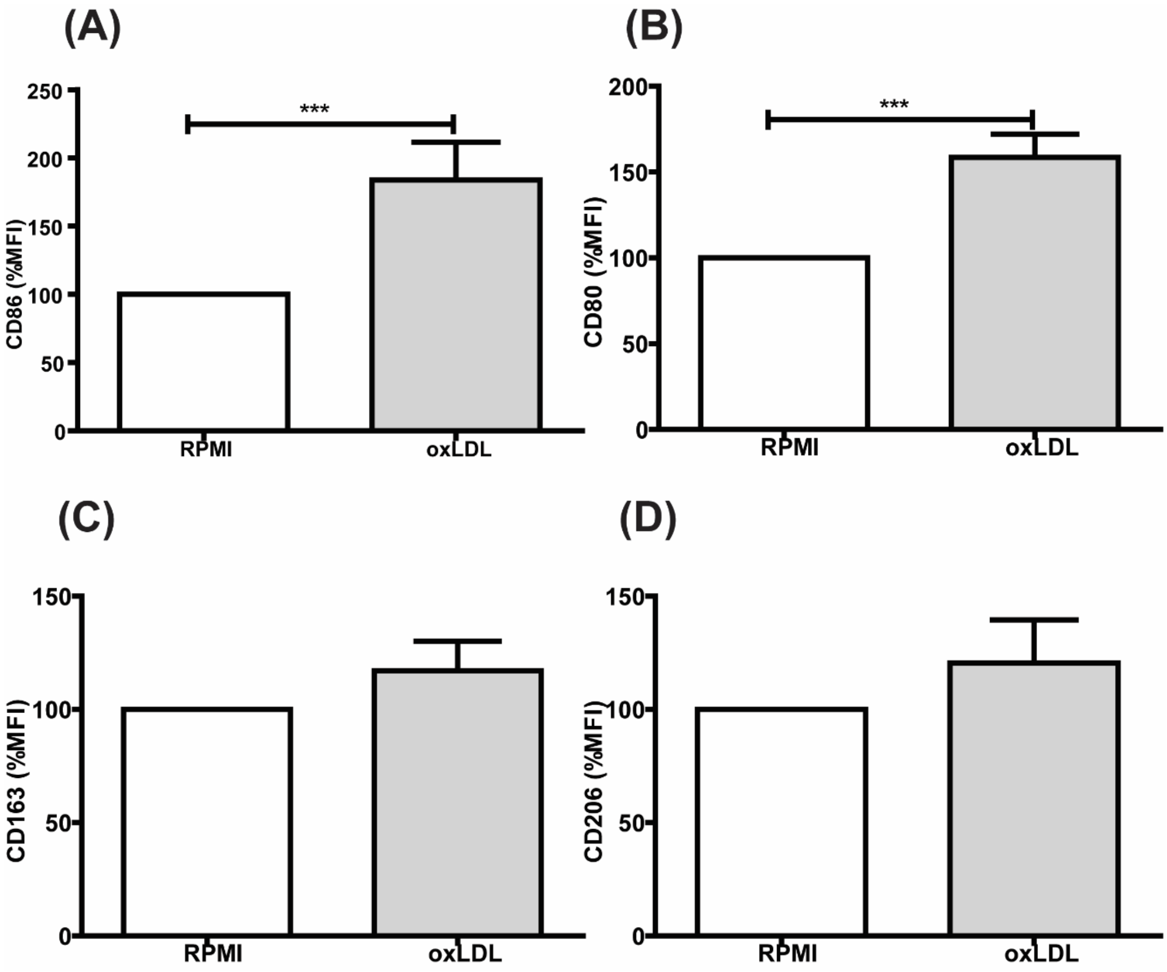

Figure S2

oxLDL treated macrophages display M1 and M2 markers. Monocytes were treated with 20µg/ml oxLDL or vehicle for 24h and rested for 5 days. On day 6 cells were harvested using ice cold PBS containing 5mM EDTA. Cells were washed with PBS, stained with surface markers for CD80-APC (Biolegend), CD86-VioBright 515 (Miltenyi Biotec), CD163-APC-Vio770 (Miltenyi Biotec) and CD206 (Biolegend) and were analyzed by FACS. (***, $P < 0.001$. Mean+SD of at least 6 samples/group from 3 different experiments were compared).

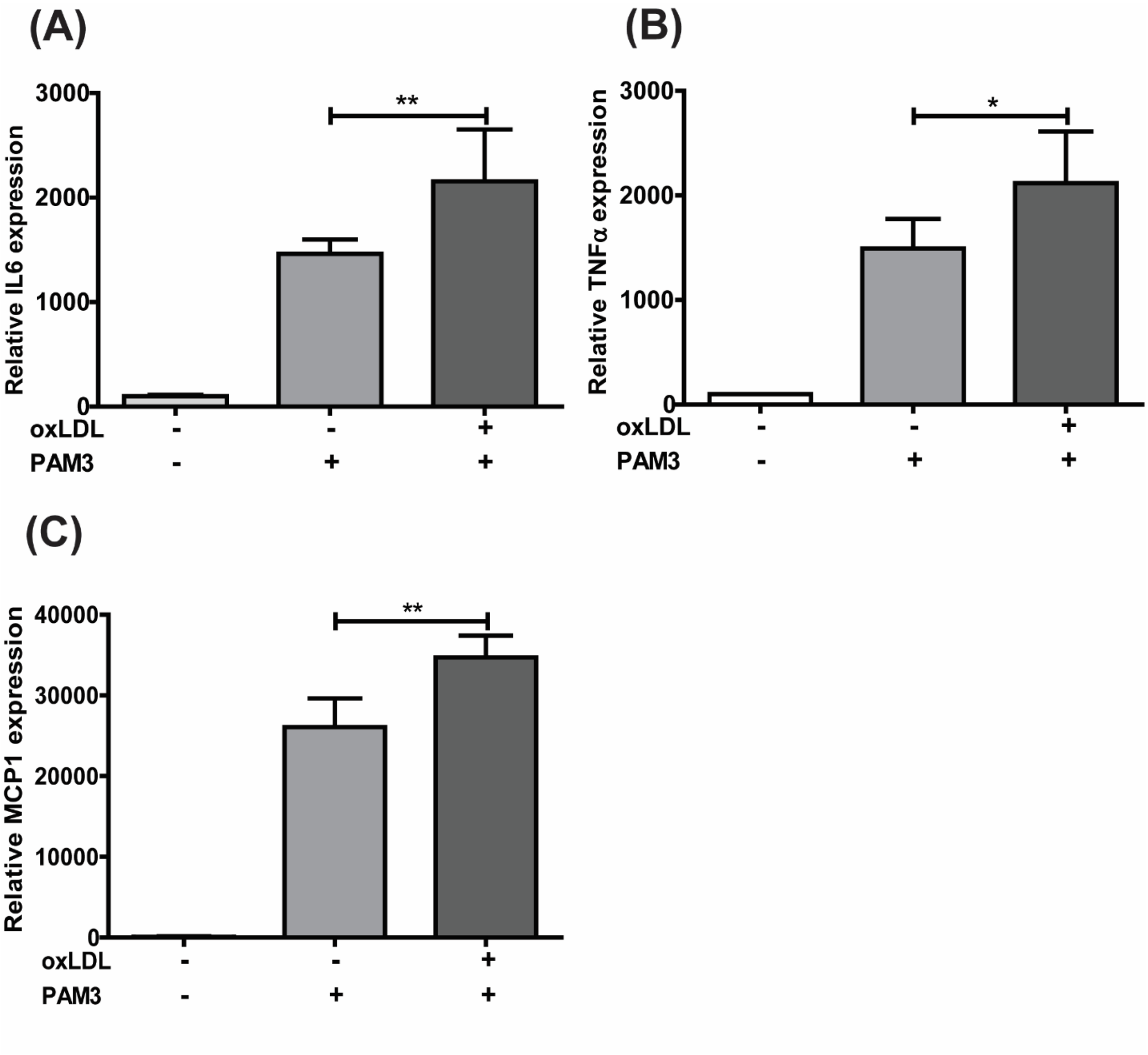

Figure S3

mRNA expression of selected inflammatory cytokines after oxLDL priming. Monocytes were treated with 20 μg/ml oxLDL or vehicle for 24h and re-stimulated with 5 μg/ml PAM3cys or vehicle for 6h on day 6. mRNA levels of *IL6*, *TNFα* and *MCP1* genes were analyzed by real-time qPCR. (*, $P < 0.05$ and **, $P < 0.01$, Mean+SD of at least 6 samples/group from 3 different experiments were compared).

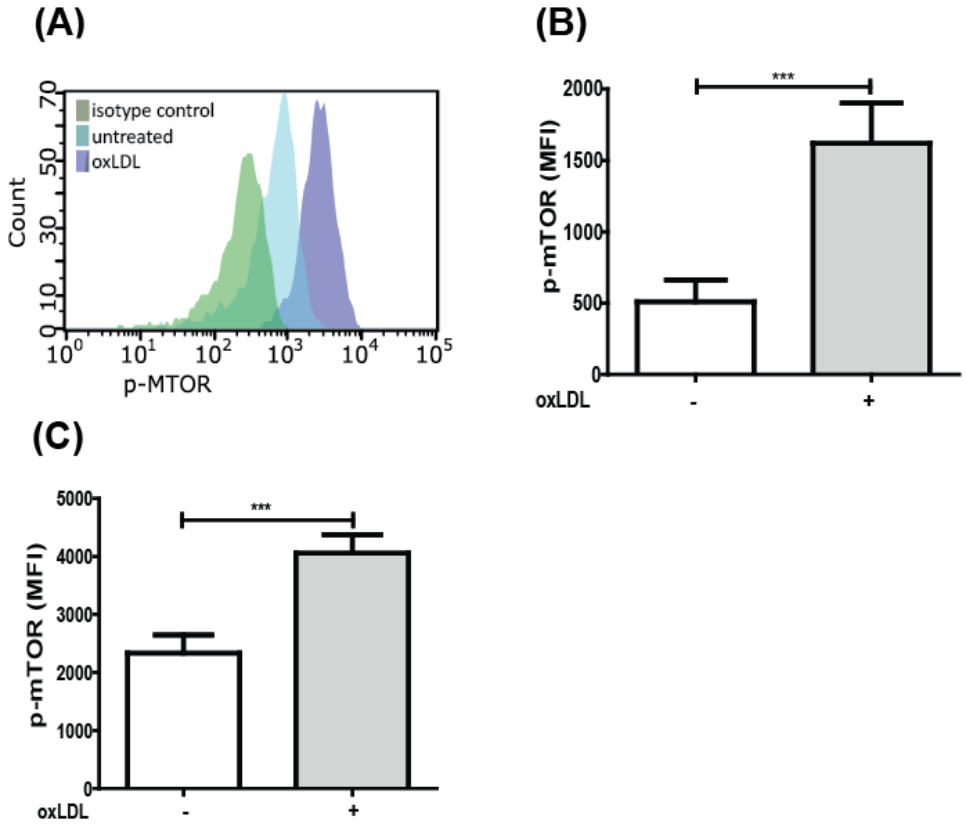

Figure S4

oxLDL induces phosphorylation of mTOR. Monocytes were treated with 20 µg/ml oxLDL or vehicle for 24h. Phosphorylation of mTOR was checked on day 1**(A, B)** and day 3**(C)**. The cells were stained with PE-Cyanine7 anti-human p-mTOR (Ser2448) and analyzed by FACS (***, P < 0.001, Mean+SD of at least 3 samples/group from 3 different experiments are shown).

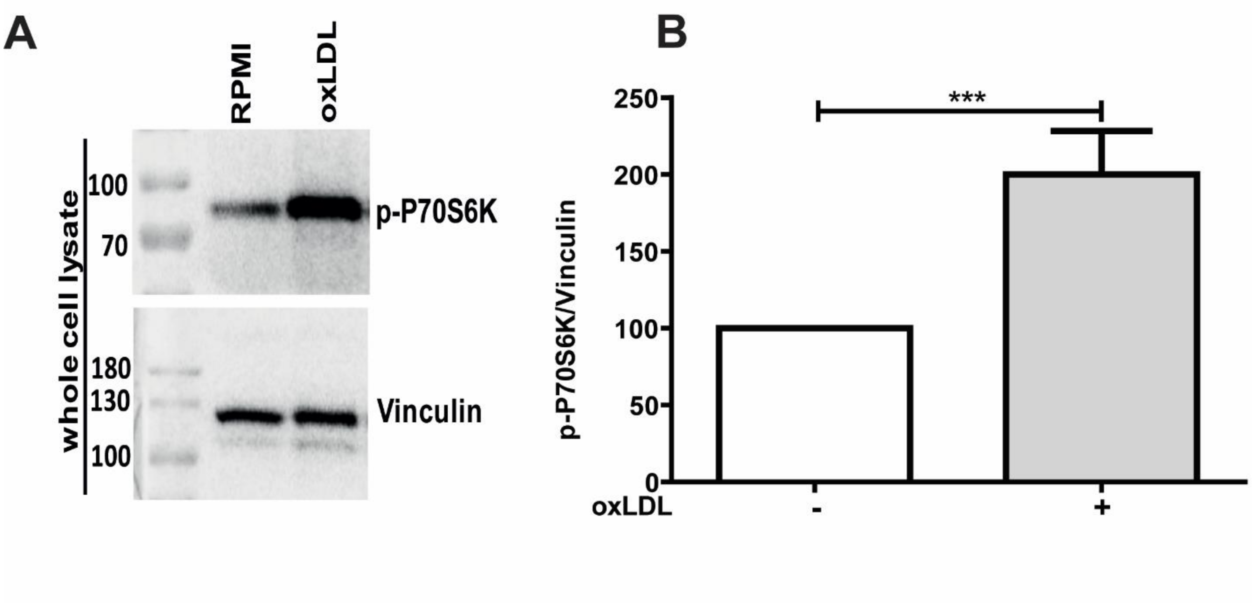

Figure S5

oxLDL induces phosphorylation of P70S6K. Monocytes were treated with 20 µg/ml oxLDL or vehicle for 24h. The cells were lysed on day 6 and stained with Phospho-p70 S6 Kinase (Thr389) Antibody (Invitrogen) and Vinculin Antibody (7F9) (Santa Cruz) and analyzed by western blot (***, $P < 0.001$, Mean+SD of at least 3 samples/group from 3 different experiments are shown).

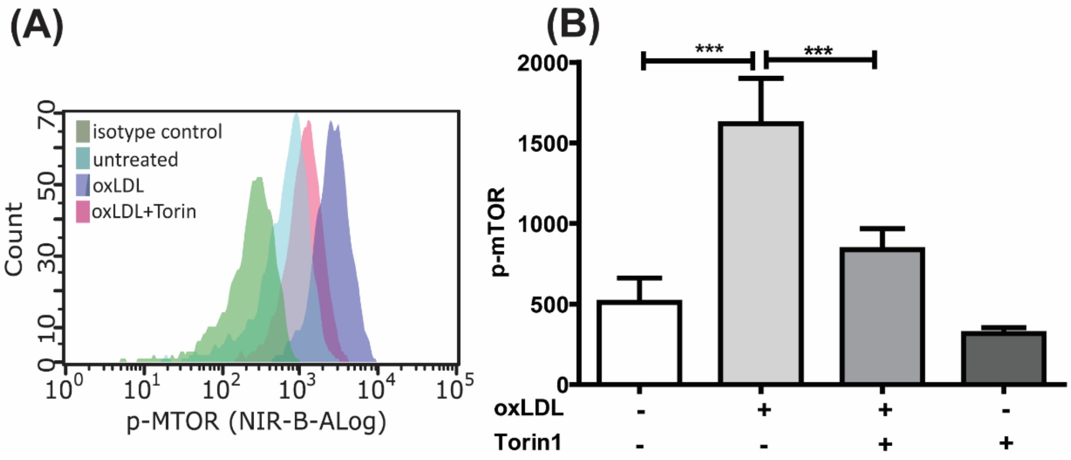

Figure S6

Torin1 inhibits phosphorylation of mTOR. Cells were treated with 100nM Torin1 or vehicle an hour before stimulation with 20 μg/ml oxLDL or vehicle. Primed cells and controls were stained with PE-Cyanine7 anti-human p-mTOR (Ser2448) (eBioscience) and analyzed by FACS. The intensity of p-mTOR was compared. Graphs represent mean values ± SD of at least 6 individuals in at least 3 different experiments (***, $P < 0.001$).

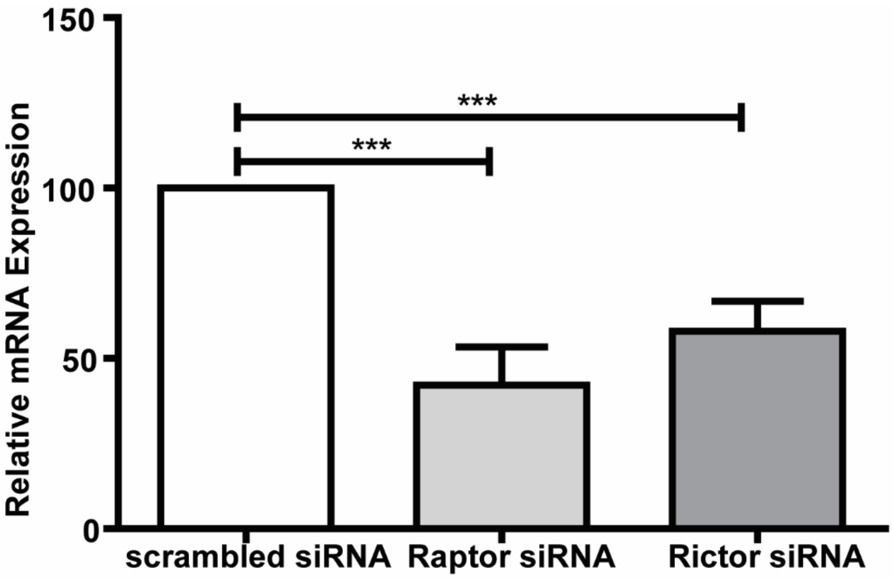

Figure S7

Gene silencing efficiencies of Raptor and Rictor siRNA. Monocytes were transfected with a final concentration of 60nm Raptor or Rictor siRNA. Cells were lysed 24h after siRNA transfection and expression level was analyzed with real-time qPCR. (***, $P < 0.001$. Mean+SD of at least 6 samples/group from 3 different experiments were compared).

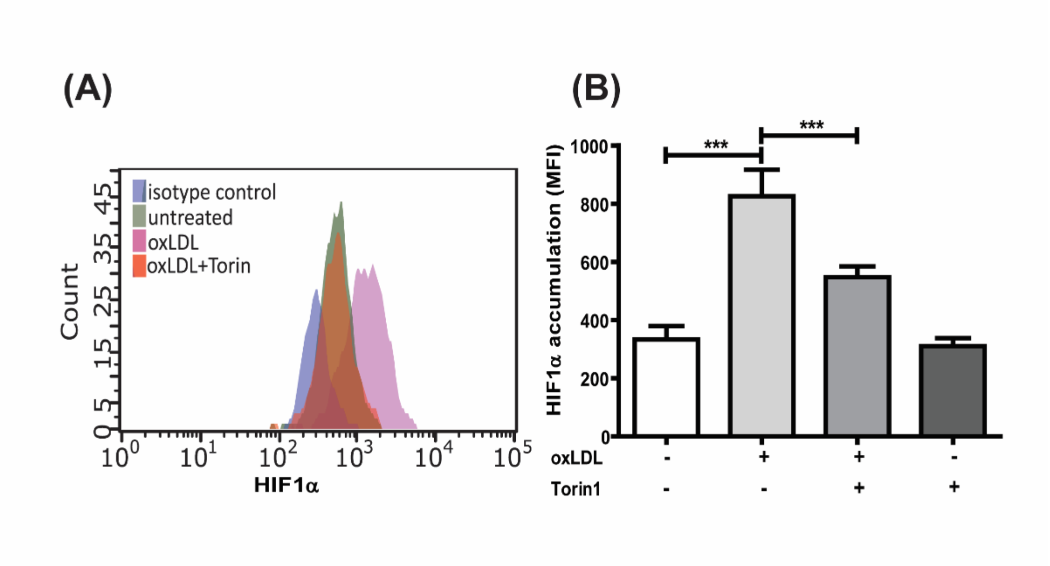

Figure S8
mTOR inhibition alters HIF1α accumulation. Cells were treated with 100nM Torin1 an hour before priming with 20 μg/ml oxLDL or vehicle for 24h. The cells were harvested on day 1 and were stained with PE anti-human HIF1α Antibody (Biolegend). The cells were analyzed by FACS and the MFI (mean fluorescence intensity) was compared. **(A)**: Representative histogram of flow cytometry analysis of HIF1α in monocytes treated with oxLDL in the presence or absence of 100nM Torin1. **(B):** Graphs represent mean values ± SD of 6 biological replicates in at least 3 different experiments. ***, $P < 0.001$.

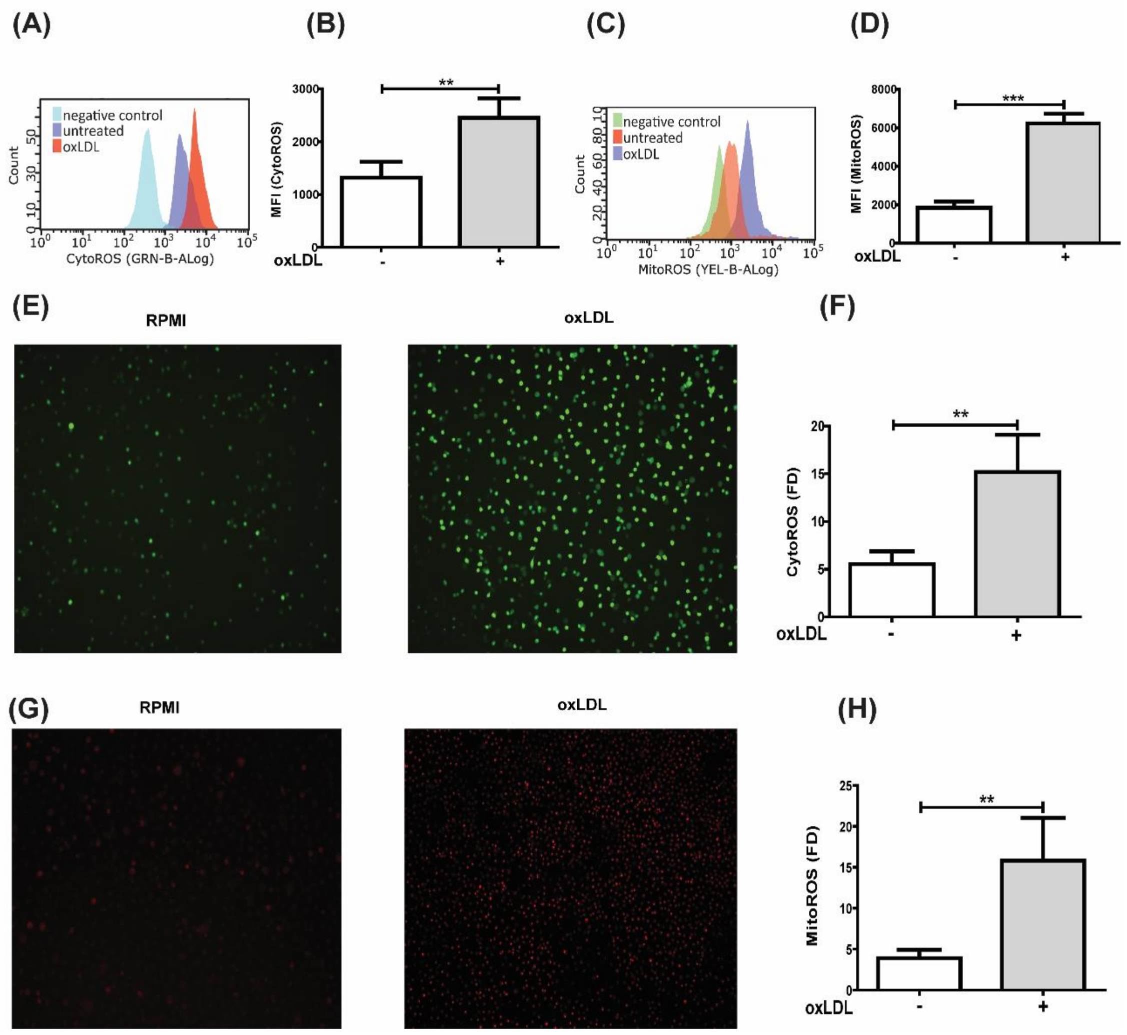

Figure S9

OxLDL induces ROS generation in monocytes. Cells were either treated with 20 μg/ml oxLDL or vehicle for 24 h and stained for cytosolic ROS (CytoROS) using 5 μM CellROX green or mitochondrial ROS (MitoROS) using 2.5μM MitoSOX red and analyzed by FACS. A: Histogram overlays and columns show intensity of CellROX (A, B) and MitoSOX (C, D). OxLDL treated and control cells were also stained with CellROX green (E, F) and MitoSOX red (G, H), checked with a microscope and quantified by ImageJ. Graphs represent mean values ± SD of at least 6 individuals in at least 3 different experiments. (**, $P < 0.01$ and ***, $P < 0.001$).

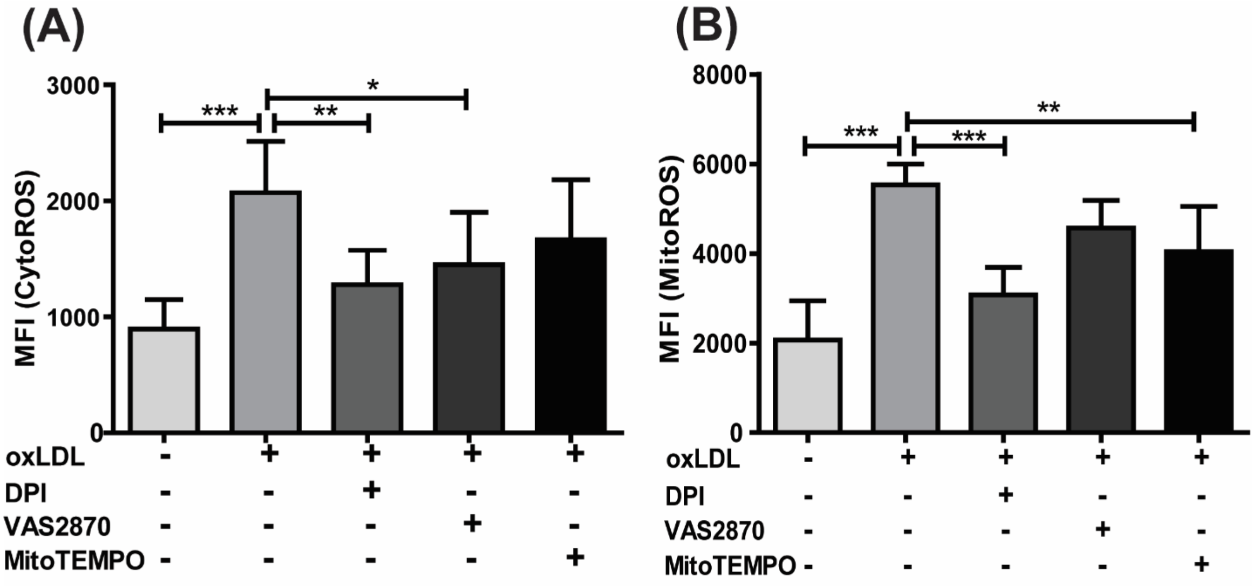

Figure S10

Antioxidant treatment inhibits ROS formation. Monocytes were treated with antioxidants 0.5 µM of Diphenyleneiodonium (DPI), 25 µM VAS2870 or 40µM mitochondrial ROS scavenger Mito-TEMPO or vehicle one hour before oxLDL priming. Cells were primed with 20 µg/ml oxLDL or vehicle and 24h after adding oxLDL cells were collected and stained with 5 µM CellROX green (A) or 2.5µM MitoSOX Red (B) and analyzed by FACS. Graphs represent mean values ± SD of at least 6 individuals in at least 3 different experiments. (*, $P < 0.05$, **, $P < 0.01$ and ***, $P < 0.001$).

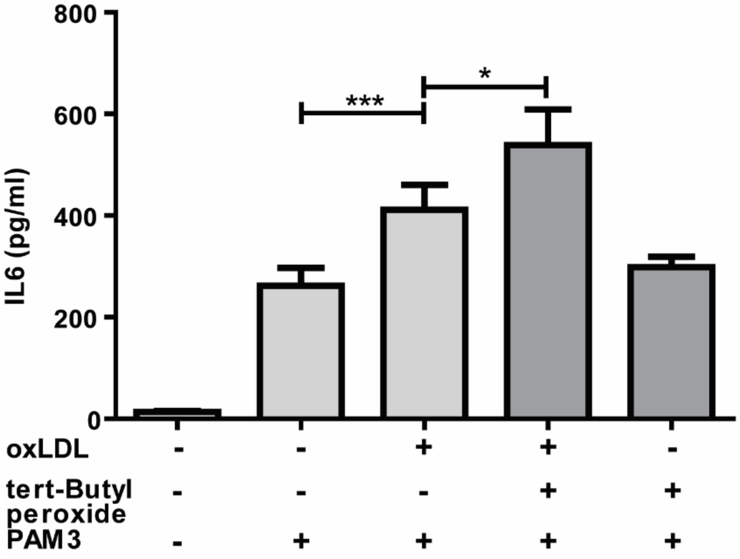

Figure S11

Increased oxidative stress enhances oxLDL priming effect. Monocytes were pretreated with 200μM tert-Butyl peroxide or vehicle before oxLDL treatment (20 μg/ml for 24h) or vehicle. Synergic effect of tert-Butyl peroxide and oxLDL in inducing trained immunity was shown by analyzing IL6 production in at least 6 individuals in at least 3 different experiments (*, $P < 0.05$ and ***, $P < 0.001$).

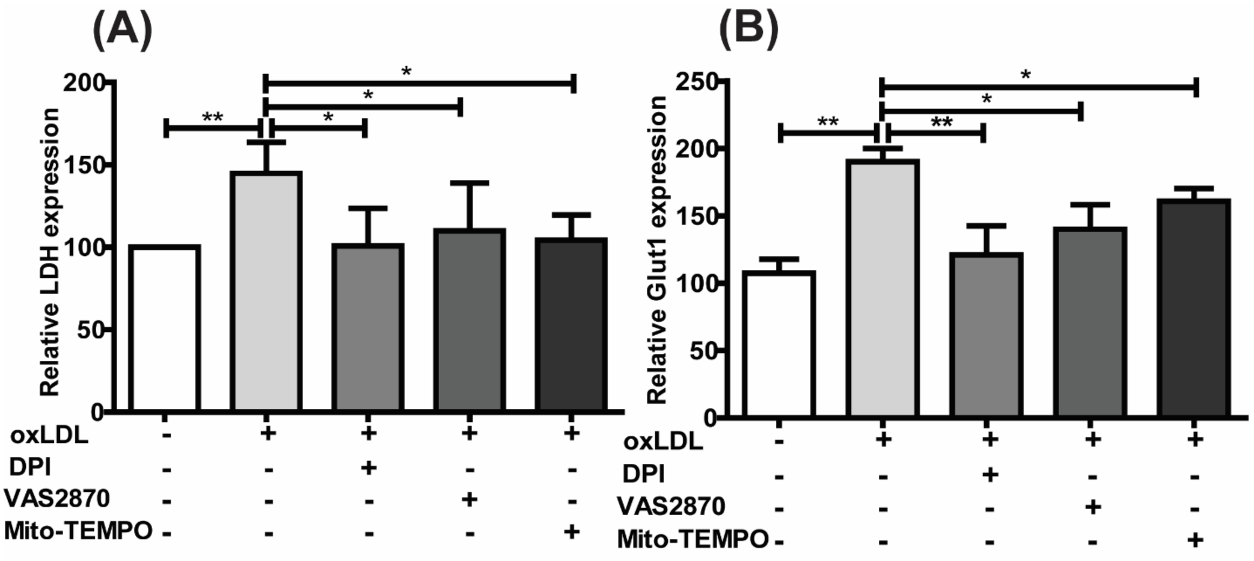

Figure S12

mRNA expression of *LDH* and *Glut1* genes after oxLDL treatment. Monocytes were treated with 20 µg/ml oxLDL or vehicle for 24h, and rested for 5 days. On day 6 cells were lysed and mRNA levels of the genes were analyzed by real-time qPCR. (*, $P < 0.05$ and **, $P < 0.01$. Mean+SD of at least 6 samples/group from 3 different experiments were compared).
